# Virtual Screening of FDA-Approved Drugs for Enhanced Binding with Mitochondrial Aldehyde Dehydrogenase

**DOI:** 10.3390/molecules27248773

**Published:** 2022-12-10

**Authors:** Boqian Zhou, Yongguang Zhang, Wanyun Jiang, Haiyang Zhang

**Affiliations:** 1 Department of Biological Science and Engineering, School of Chemistry and Biological Engineering, University of Science and Technology Beijing, Beijing 100083, China; 2 School of Life Sciences, Beijing Institute of Technology, Beijing 100081, China

**Keywords:** virtual screening, drug repurposing, molecular dynamics simulation, alcohol addiction

## Abstract

Mitochondrial aldehyde dehydrogenase (ALDH2) is a potential target for the treatment of substance use disorders such as alcohol addiction. Here, we adopted computational methods of molecular dynamics (MD) simulation, docking, and molecular mechanics Poisson–Boltzmann surface area (MM-PBSA) analysis to perform a virtual screening of FDA-approved drugs, hitting potent inhibitors against ALDH2. Using MD-derived conformations as receptors, butenafine (net charge *q* = +1 *e*) and olaparib (*q* = 0) were selected as promising compounds with a low toxicity and a binding strength equal to or stronger than previously reported potent inhibitors of daidzin and CVT-10216. A few negatively charged compounds were also hit from the docking with the Autodock Vina software, while the MM-PBSA analysis yielded positive binding energies (unfavorable binding) for these compounds, mainly owing to electrostatic repulsion in association with a negatively charged receptor (*q* = −6 *e* for ALDH2 plus the cofactor NAD^+^). This revealed a deficiency of the Vina scoring in dealing with strong charge–charge interactions between binding partners, due to its built-in protocol of not using atomic charges for electrostatic interactions. These observations indicated a requirement of further verification using MD and/or MM-PBSA after docking prediction. The identification of key residues for the binding implied that the receptor residues at the bottom and entrance of the substrate-binding hydrophobic tunnel were able to offer additional interactions with different inhibitors such as π-π, π-alkyl, van der Waals contacts, and polar interactions, and that the rational use of these interactions is beneficial to the design of potent inhibitors against ALDH2.

## 1. Introduction

Acetaldehyde, a toxic product of ethanol metabolism mainly in the liver, is a critical factor responsible for, for instance, alcohol-induced liver damage, DNA damage, and several cancers [1,2,3,4,5,6]. Aldehyde dehydrogenases (ALDHs) are the most important enzymes catalyzing the metabolism of various reactive aldehydes to the corresponding non-toxic carboxylic acids and their derivatives [7]. The human ALDH superfamily has 19 NAD(P)^+^-dependent members with similar but not identical functions owing to their differences in gene expression and substrate specificity [8,9]. Among them, three closely related enzymes with an amino acid sequence identity of 68% (ALDH1A1, ALDH1B1 and ALDH2) were reported to be most relevant to acetaldehyde metabolism [9,10,11]. A mitochondrial isomer of ALDH2 was the most important one and prevented the accumulation of acetaldehyde by oxidizing it to acetate [12]. This enzyme was also of relevance to the reaction of other substances such as short-chain aliphatic, aromatic, and polycyclic aldehydes [13] and to a number of human pathologies such as substance use disorders (such as drug addiction) [14,15,16,17].

Alcohol use disorder (AUD), commonly known as alcohol addiction, is a leading and ubiquitous risk for personal death and disability as well as social stability. World Health Organization (WHO) reported an estimated 3 million deaths globally in 2016 from the harmful use of alcohol, accounting for 5.3% of all deaths [18]. In China, about one ninth of middle-aged men suffered from AUD [19]. A semi-dominant polymorphism of the ALDH2 gene, ALDH2*2, exists in ca. 8% of the world’s population, 40% of which are Asian [20,21,22,23]. ALDH2*2 encodes a nearly inactive protein and the reduced activity retards acetaldehyde metabolism, leading to a high level of acetaldehyde in the blood after alcohol intake and thus to discomforts such as facial flushing, nausea, and palpitation [20]. Such polymorphism provided a protective effect against alcoholism and alcohol-induced diseases, which greatly decreased the risk of alcohol dependence or abuse [24,25].

When an inhibitor reduced the activity of ALDH2, the level of alcohol-induced dopamine in the nervous system was shown to be downregulated [26], and similar observations were detected for the exposure to methamphetamine and cocaine [15,16]. ALDH2 inhibition is therefore a therapeutic strategy for the treatment of AUD and drug addiction. Four compounds were approved for treating alcohol addiction: disulfiram, acamprosate, and naltrexone by the Food and Drug Administration (FDA) in the United States, and nalmefene by the European Medicines Agency (EMA) [27]. Of these compounds, only disulfiram targets ALDHs; however, it is nonspecific and is able to inhibit a variety of receptors such as ALDH1, ALDH2, dopamine β-hydroxylase, and phosphoglycerate dehydrogenase [28,29,30]. The design of specific and selective inhibitors against ALDH2 is therefore highly desirable for the treatment of substance use disorders. Isoflavone analogues of daidzin [31] and CVT-10216 [26] were verified in the experiments as potent inhibitors of ALDH2 with IC_50_ values of 0.08 and 0.029 μM, respectively. In a virtual screening of a commercial chemical library with 50,000 compounds, Wang et al. identified five small-molecule inhibitors, four of which had an IC_50_ of 0.5–3.8 μM [32].

Drug repurposing is a promising and productive approach to exploring old drugs for new use, which accelerates the process of drug discovery [33]. FDA-approved drugs constitute an ideal database for such a purpose. A variety of compounds, for instance, were hit from this database for a possible treatment of virus infection such as Zika [34] and SARS-CoV-2 [35,36,37] and for inhibition against disease-related targets such as arginases [38], N-acetyltransferase 10 [39], eukaryotic elongation factor-2 kinase [40], and triosephosphate isomerase [41]. However, repurposing FDA-approved drugs for inhibition against ALDH2 is rarely reported. Here, we aimed to select potential inhibitors from the FDA-approved drug database with enhanced binding with ALDH2. Structural flexibility in the receptor–inhibitor binding was taken into account by all-atom molecular dynamics (MD) simulations. We also evaluated the toxicity of the selected drug compounds and decomposed the binding energies to identify key residues. Interaction types and the effects of net charges of inhibitors on the binding were discussed at the end of the manuscript as well as the selectivity of hit compounds for the human ALDH family members. We believe this work is valuable for the further design of potent inhibitors against ALDH2. 

## 2. Results and Discussion

### 2.1. MD Simulation of ALDH2 Tetramer

**Figure 1:**
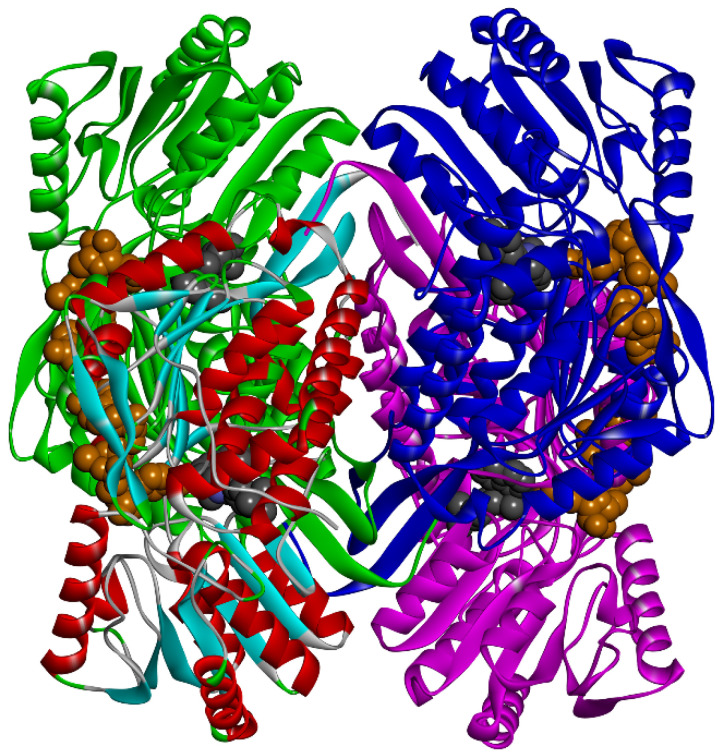
ALDH2 tetramer in complex with the cofactor NAD^+^ and potential inhibitor butenafine. Proteins are represented with the solid ribbon model; chain A is colored by secondary structures (red for *α*-helix and cyan for *β*-sheet), while chains B, C, and D are in green, blue, and magenta, respectively. NAD^+^ (orange) and inhibitor (dark grey) molecules are displayed using the space-filling model (CPK).

**Figure 2:**
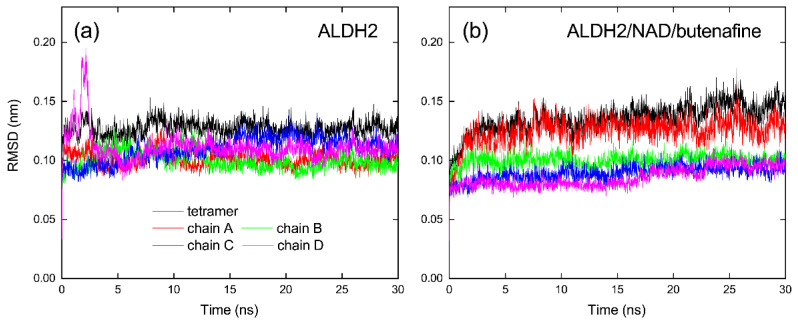
Root-mean-square deviation (RMSD) of protein backbone from crystal structure as a function of simulation time for the ALDH2 tetramer and monomers (chains A–D) in the apo form (**a**) and in the complex form (**b**) with the co-factor NAD^+^ and the inhibitor butenafine.

Equilibration of ALDH2 tetramer in MD simulations with explicit solvent molecules allowed considering the structural flexibility of receptors in the virtual screening of ligand compounds. ALDH2 was crystalized experimentally in a tetramer form [42], and interfacial residues between monomers affected the structural stability of catalytic sites and cofactor binding domains [22,23,43,44], as presented in Figure 1. Simulation of a dimer was reported to be capable of maintaining the ALDH2 stability in a previous study [45]. Here, we chose to simulate the whole tetramer in water. Root-mean-square deviation (RMSD) of ALDH2 backbone atoms from the crystal structure tended to be stable and converged to a value smaller than 0.13 nm during the MD simulations with a length of 30 ns (Figure 2a), indicating that the ALDH2 structure was well maintained in the apo form. The RMSDs of the monomers (chains A–D) showed that there was a slight difference in the structural stability of different monomers. From a cluster analysis on the simulation of ALDH2 tetramer, we obtained 50 representative structures of the ALDH2 monomer for the following docking calculations.

### 2.2. Virtual Screening of FDA-Approved Drugs against ALDH2

The crystal structure (chain A) and MD-derived snapshots of ALDH2 were adopted as receptors (51 in total) for the virtual screening of FDA-approved drugs (2115 ligands in total) using the Autodock Vina software [46]. The receptor ALDH2 (monomer) contained two binding domains for association with the substrate (such as acetaldehyde or inhibitor) and the cofactor NAD^+^, respectively, and both domains were adjacent to each other, as shown in Figure 1. This necessitated the existence of NAD^+^ in the docking; if not, the ligand would enter into the cofactor-bound cavity.

The top 10 drugs from virtual screening using the crystal structure of the receptor were tabulated in Table 1 in ascending order of binding affinities with ALDH2, as well as their structures and formal charges. Differin (ZINC ID: ZINC003784182) showed the strongest binding affinity (∆*E*_dock_) of −48.5 kJ/mol, while the 10th drug amaryl (ZINC000537791) gave a value of −43.5 kJ/mol. Binding affinities of the top ten drugs were equal to or more negative than the previously reported inhibitors of CVT-10216 (∆*E*_dock_ = −43.5 kJ/mol) and daidzin (∆*E*_dock_ = −38.1 kJ/mol) [45]. Both reported inhibitors belong to isoflavone analogues and showed a strong inhibition against ALDH2 with IC_50_ values of 0.029 and 0.08 μM for CVT-10216 [26] and daidzin [42], respectively. This finding implied that it was possible to find a compound with enhanced binding with ALDH2 from the FDA-approved drug database.

Using MD-derived structures as receptors, the virtual screening resulted in more hits with binding affinities (∆*E*_dock_) stronger than −46.4 kJ/mol (Table 2). Mepron showed the highest binding affinity with ALDH2 (∆*E*_dock_ = −51.1 kJ/mol). Two isomers of noxafil, ZINC028639340 and ZINC003938482, yielded different ∆*E*_dock_ of −47.7 and −47.3 kJ/mol, respectively. Differin (ZINC003784182) and eltrombopag (ZINC011679756) were hit again, with an identical binding affinity with both crystal and MD-derived structures of ALDH2 (Table 1 and Table 2). Interestingly, different charge states of eltrombopag (−3 *e* in Table 1 and −2 *e* in Table 2) were selected, although there was a slight difference in the used receptor structures. This implied that the Vina scoring was likely not capable of evaluating different charge states with good accuracy. In total, six compounds of the top 10 drugs in both Table 1 and Table 2 were not neutral and had a positive or negative net charge. These charged compounds therefore needed to be treated with care.

### 2.3. Toxicity Evaluation

The toxicity of the selected compounds were evaluated by ProTox-II [47]. Hepatotoxicity is of virtual importance because liver is the primary organ where the metabolism of ethanol and other drugs takes place [48,49]. There were 17 hits from FDA-approved drugs with potential inhibition against ALDH2, of which eight compounds were predicted to be active for the organ toxicity (hepatotoxicity; dili for short in Table 1 and Table 2) with a confidence score of ≥0.67. These compounds were excluded from further evaluation. The carcinogenicity of differin and fexofenadine was predicted with a confidence score of 0.61 and 0.50, respectively, whereas it was inactive for the other compounds. Indacaterol, orap, and thalitone probably had adverse effects on the immune system (immunotoxicity) with a confidence score of ≥0.80. All of the selected drugs appeared inactive for mutagenicity and cytotoxicity, except stivarga, likely causing cell damage (cytotoxicity) with a confidence score of 0.77. CVT-10216, a potent inhibitor of ALDH2 [15,26], was predicted active for hepatotoxicity and mutagenicity with a relatively low confidence score of 0.56 and 0.54 (Table 1), respectively. As a naturally occurring isoflavone, daidzin was inactive for all of the tested toxicities with a confidence score of 0.59–0.85 (Table 1).

Based on the toxicity evaluation, mepron (*q* = −1 *e*), differin (−1 *e*), olaparib (neutral), butenafine (+1 *e*), fexofenadine (neutral), montelukast (−1 *e*), and amaryl (−1 *e*) were selected as inhibitor candidates with potent binding with ALDH2 and almost no toxicity. The first five ones were further investigated by MD simulations for the association with ALDH2, and daidzin (neutral) was simulated as well for comparison.

### 2.4. MD Simulation of ALDH2–Inhibitor Complexes and Binding Energy Calculation

**Figure 3:**
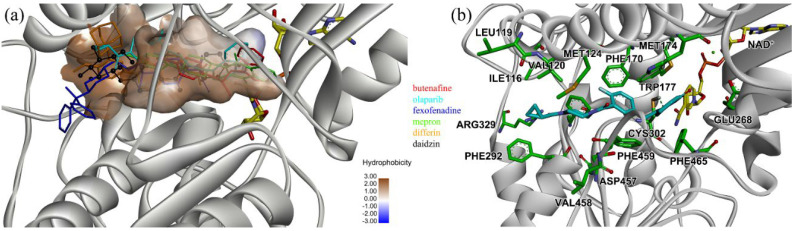
(**a**) Binding poses of ALDH2 with selected inhibitors from docking and (**b**) representative binding mode of ALDH2 with olaparib from MD simulations. Five compounds (butenafine, olaparib, fexofenadine, mepron, and differin) are chosen as potential inhibitors and are shown with the stick models in different colors (**a**). The pose of daidzin (colored in black) in the crystal structure (PDB code: 2VLE) is displayed with the scaled ball and stick model for reference (**a**). The hydrophobic surface on the receptor is created using the ALDH2–daidzin binding pose to depict the hydrophobic tunnel for ligand binding (**a**). Carbon atoms of olaparib in the complex with ALDH2 are colored in cyan, while the carbon atoms of interacting receptor residues are in green (**b**). The cofactor NAD^+^ is shown by the stick model with its carbon atoms in yellow, and the green ball in the panel (**b**) is Mg^2+^ ion bound to NAD^+^. Hydrogen atoms are removed for clarity.

Docking poses of the receptor ALDH2 with the inhibitors were used as initial configurations for the MD simulations of ALDH2 tetramers in the complex form, as shown in Figure 3a for one monomer (chain A). For a clear depiction of ligand binding, we highlighted a hydrophobic surface on the receptor using the ALDH2/daidzin complex; the isoflavone skeleton (hydrophobic) was buried in the hydrophobic tunnel, while its glucose group (hydrophilic) lied at the entrance of the tunnel (Figure 3a). The inhibitors penetrated into the hydrophobic tunnel of ALDH2, while the penetration depth differed from case to case. Compared with daidzin, it appeared that butenafine, olaparib, and mepron could go further and lie at the bottom of the tunnel; however, the penetration of fexofenadine and differin was much shallower, and their hydrophobic moieties such as the benzene ring of fexofenadine or adamantane group of differin were located outside of the tunnel. This implied that the hydrophobic tunnel of ALDH2 for ligand binding was larger than the isoflavone skeleton in length, in line with experimental [31] and computational [45] observations.

Root-mean-square deviation (RMSD) of protein backbone atoms from the crystal structure during 30 ns MD simulation of the ALDH2/butenafine/cofactor complex is presented in Figure 2b. Association with butenafine and NAD^+^ produced RMSD values of 0.13 and 0.15 nm for the monomer A and tetramer, respectively, larger than that in the ligand-free (apo) form of ALDH2 (Table 3). The other three monomers (chains B–D) showed a RMSD of ca. 0.1 nm, very similar to the structural fluctuations of the apo form. Similar observations were detected for the complexes with the inhibitors of fexofenadine and mepron (Table 3). For all of the tested inhibitors, crystal structures of chains B-D in the ALDH2 tetramer appeared more stable than chain A, as indicated by the smaller RMSD values (Table 3). Despite these differences, protein structures of the ALDH2 monomers and tetramer tended to be stable after 20 ns simulation (Figure 2b), and the last 10 ns trajectories were therefore used for data analysis.

Receptor-ligand binding energies (Δ*E*_bind_) for each monomer were computed from the Molecular mechanics Poisson–Boltzmann surface area (MM-PBSA) analysis [50]. Significant differences were observed in different monomers (Table 3). Upon association with fexofenadine, for instance, monomers A and C yielded a value of Δ*E*_bind_ ≈ −35 kJ/mol, whereas monomers B and D produced a value of ca. −90 kJ/mol. For a quantitative evaluation, binding energies of four monomers were averaged with a weight of their Boltzmann factors (Equation (1)) [51,52].

(1) $$\langle ∆E_{\mathrm{bind}}\rangle =\frac{\sum_{i}∆E_{\mathrm{bind},i}\exp\left(-∆E_{\mathrm{bind},i}/RT\right)}{\sum_{i}\exp\left(-∆E_{\mathrm{bind},i}/RT\right)}$$

where *i* is the monomer ID (chains A–D), *R* is the ideal gas constant, and *T* is the absolute temperature (298.15 *K*). The weighted binding affinities <Δ*E*_bind_> were very close to the most negative value among different monomers. That is, the ALDH2 monomer B outperformed the other monomers for association with fexofenadine, mepron, and differin, while it was the monomer C for butenafine, olaparib, and daidzin. These monomers with good performances were used for the following energy decomposition to explore the details of the interactions between binding partners.

Binding energy (Δ*E*_bind_) was decomposed into four components of van der Waals (Δ*E*_vdW_) and electrostatic interactions (Δ*E*_elec_) as well as polar (Δ*G*_polar_) and nonpolar (Δ*G*_nonpolar_) solvation contributions. The sum of the first two contributions was the so-called MM part (Δ*E*_MM_); see Equation (2) in the Section 3.4 for details on the decomposition. Δ*E*_vdW_ and Δ*G*_nonpolar_ favored the binding of ALDH2 with inhibitors, while Δ*G*_polar_ disfavored the complexation (Table 4). For neutral and positively charged inhibitors, Δ*E*_elec_ had a favorable contribution; for butenafine with a net charge of +1 *e*, in particular, a strong electrostatic interaction was measured with a contribution of about −280 kJ/mol (Table 4). This was likely ascribed to the electrostatic attraction of the negatively charged receptor (−6 *e* for ALDH2 monomer). Due to the repulsion of charges with like sign, unfavorable contributions of electrostatic interactions were observed for the negatively charged inhibitors of mepron and differin, as indicated by positive Δ*E*_elec_ of ca. 100 and 75 kJ/mol, respectively (Table 4). We did not carry out such an analysis for montelukast and amaryl with a net charge of −1 *e* (Table 1 and Table 2), and as stated above, these two compounds probably produced unfavorable binding with ALDH2.

The MM part (Δ*E*_MM_) favored the inhibitor binding owing to a relatively large contribution of Δ*E*_vdW_. Solvation energies (Δ*G*_sol_) displayed unfavorable contributions of roughly 110–300 kJ/mol, probably arising from the desolvation of the binding partners. Electrostatic repulsions between ALDH2 and the inhibitors of mepron and differin resulted in a Δ*E*_MM_ contribution of −125 kJ/mol, and the relatively large Δ*G*_sol_ gave rise to positive binding energies (Δ*E*_bind_) for these two compounds (Table 4). Electrostatic attractions endowed butenafine with a very strong binding against ALDH2 (Δ*E*_bind_ = −313.4 kJ/mol). Δ*E*_bind_ for daidzin was −110.1 kJ/mol, close to our previous prediction of −105.8 kJ/mol on the ALDH2 dimer [45]. Fexofenadine (Δ*E*_bind_ = −94.4 kJ/mol) showed a weaker binding with ALDH2 than daidzin. The neutral drug of olaparib yielded a Δ*E*_bind_ of −127.6 ± 8.7 kJ/mol (Table 4), a binding affinity similar to the potent inhibitor of CVT-10216 (Δ*E*_bind_ = −132.3 ± 7.6 kJ/mol) [45]. These findings indicated that butenafine (*q* = +1 *e*) and olaparib (*q* = 0) were ideal inhibitors with enhanced binding with ALDH2 compared to daidzin and CVT-10216.

Note that our virtual screening with molecular docking predicted equal or more negative binding affinities by 2–8 kJ/mol for the five selected drugs than CVT-10216, as indicated by ∆*E*_dock_ in Table 1 and Table 2. However, the MM-PBSA analysis produced a very strong binding for the positively charged butenafine and an unfavorable binding (i.e., positive Δ*E*_bind_) for the negatively charged mepron and differin. This was ascribed to the Vina scoring not using atomic charges for electrostatic interactions. As designed, it was not necessary to assign atomic charges for the Autodock Vina software and electrostatic interactions were handled via hydrophobic and hydrogen bonding terms with empirical parameters [46]. Therefore, it could be an issue in the modeling of strong electrostatic interactions between charged moieties in the Vina scoring. The MM-PBSA analysis might provide a solution, although it required relatively heavy computational loads.

### 2.5. Identification of Key Residues for Receptor-Inhibitor Interactions

**Figure 4:**
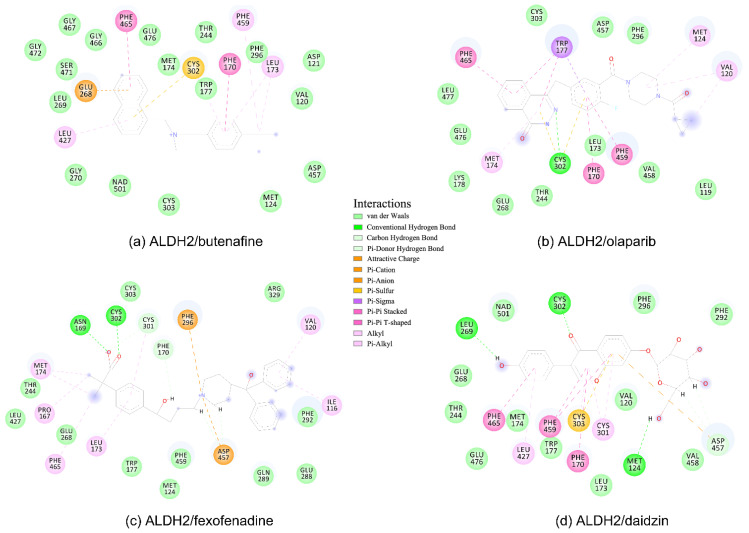
2D diagrams of receptor–ligand interactions for ALDH2 complexes with butenafine (**a**), olaparib (**b**), fexofenadine (**c**), and daidzin (**d**). The cofactor NAD^+^ is treated as one residue of the receptor with an ID of 501. The left moieties of the inhibitors enter into the bottom of the ALDH2 substrate-binding tunnel, while the right parts lie in the tunnel entrance (Figure 3a). Averaged structures from the last 10 ns simulations were used to generate the diagrams, and interacting residues and interaction types are shown with different colors.

Four compounds of butenafine, olaparib, fexofenadine, and daidzin with potential inhibition against ALDH2 were used to explore the interaction mechanism between the binding partners. The 2D diagrams of receptor–inhibitor interactions for these compounds were presented in Figure 4, where the left moieties of the inhibitors were inserted into the hydrophobic tunnel of the receptor. A number of interaction types were responsible for the complexation, namely, vdW contacts, hydrogen bonds, charge attractions and benzene-involved interactions of π-ion, π-sulfur, π-σ, π-alkyl, and π-π stacking, as well as the interactions between alkyl groups. Representative binding mode of ALDH2 with olaparib was shown in Figure 3b for a clean illustration of the substrate-binding domain and the location of interacting residues.

**Figure 5:**
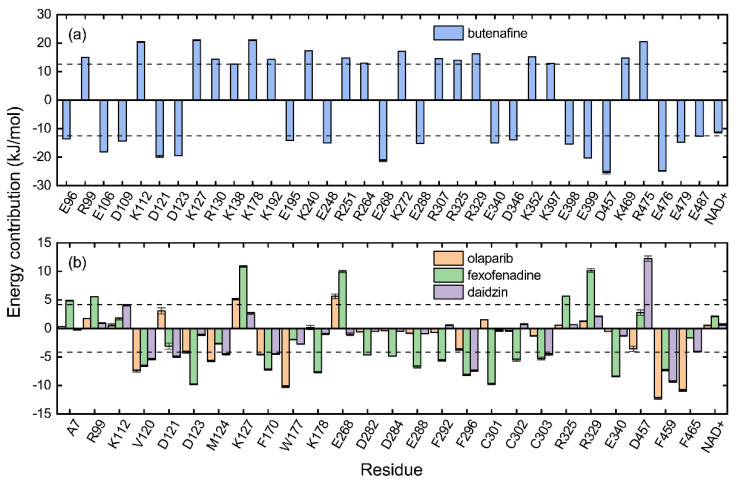
Energy contribution per residue to the binding of ALDH2 with potential inhibitors of butenafine, olaparib, fexofenadine, and daidzin. Each residue with a contribution of ≥16.8 kJ/mol is given for butenafine (**a**). For the other three compounds, the given residues have a contribution of ≥4.2 kJ/mol for at least one inhibitor (**b**). Dashed lines in panels (**a**,**b**) indicate the energy thresholds for key residue identifications. The cofactor NAD^+^ is considered as one residue of the receptor and its contribution is presented as well.

In order to identify key residues for receptor–inhibitor interactions, binding energies from the MM-PBSA analysis were further decomposed into contributions per residue. ALDH2 monomer had 500 amino acids, and the cofactor NAD^+^ with a net charge of −1 *e* was regarded as one residue of the receptor with an index of 501. Since butenafine displayed a very strong binding, only the receptor residues with a contribution of ≥16.8 kJ/mol (ca. 4 kcal/mol) were marked, which resulted in 36 key residues. For the other three compounds, the 27 key residues contributed more than 4.2 kJ/mol (ca. 1 kcal/mol) for at least one inhibitor. Energy contributions per residue to the receptor–inhibitor association are given in Figure 5 and Tables S1–S4 in the Supplementary Material.

The key residues for butenafine (*q* = +1 *e*) were composed of positively (Arg and Lys) and negatively (Asp, Glu, and NAD) charged residues, offering unfavorable (electrostatic repulsion) and favorable (electrostatic attraction) interactions, respectively (Figure 5a). Butenafine had a positively charged center (N^+^, Figure 4a); electrostatic repulsion and attraction interaction belonged to long-range interactions and no direct contacts were observed between the charged center and receptor residues in the 2D interaction diagram (Figure 4a). Fexofenadine was a neutral compound, while it had two charged moieties. The negatively charged center (COO^−^) hydrogen bonded with Asn169 and Cys302, and the positively charged center (N^+^) provided a π-cation interaction with Phe296 and a charge attraction with Asp457 (Figure 4c). For the binding of fexofenadine with ALDH2, Phe296 and Cys302 contributed favorable interactions of −8.1 and −5.5 kJ/mol, whereas Asn169 and Asp457 only had small contributions of −1.1 and 2.8 kJ/mol, respectively (Figure 5b).

Phe465 was located at the bottom of substrate-binding tunnel, and offered π-π interactions with the left benzene rings of butenafine, olaparib, and daidzin (Figure 4, panels a, b, and d, respectively). Due to a relatively shallow penetration, Phe465 interacted the alkyl group of fexofenadine (Figure 4c). This residue contributed −6.0, −10.9, −1.7, and −4.1 kJ/mol to the complexation with butenafine, olaparib, fexofenadine, and daidzin, respectively (Figure 5). Another residue of Trp177 at the tunnel bottom offered vdW contacts, π-σ, and/or π-π interactions with butenafine and olaparib, leading to favorable contributions of −7.7 and −10.2 kJ/mol (Figure 5), respectively. Owing to the difference in the penetration depth (Figure 3a), Trp177 provided vdW contacts only with fexofenadine and daidzin (Figure 4) and yielded a small contribution of ca. −2 kJ/mol.

Phe170 and Phe 459 lied roughly in the center and on both sides of the tunnel (Figure 3b), and they were therefore able to interact with the central hydrophobic moieties of the inhibitors such as benzene rings and alkyl chains (Figure 4). Phe170 produced a favorable contribution of −6 kJ/mol on average, while Phe465 contributed −12.3 kJ/mol when interacting with olaparib and ca. −8 kJ/mol for the other compounds. Cys302, close to the bottom of the tunnel, was capable of hydrogen bonding with olaparib, fexofenadine, and daidzin and/or offering π-sulfur interactions with butenafine and olaparib (Figure 4). The hydrogen bond between Cys302 and the carboxyl group of fexofenadine showed a contribution of −5.5 kJ/mol, whereas it was negligible when bound to olaparib and daidzin (Figure 5b).

The residues at the entrance of the tunnel provided hydrophobic contacts (such as Ile116, Leu119, Val120, Phe292, and Val458) or hydrophilic interactions (such as Asp457 with hydrogen bonding or charge attraction), as presented in Figure 3b. Asp457 produced a favorable Δ*E*_MM_ for all of the four inhibitors (Tables S1–S4); however, owing to the difference in the Δ*G*_sol_, this residue favored the binding for butenafine and olaparib and showed the opposite for fexofenadine and daidzin (Figure 5). In addition, the two benzene rings of fexofenadine interacted with polar residues such as Glu288, Gln289, and Arg329 via vdW contacts. These observations showed that the tunnel entrance might provide much more interactions for ligand complexation than that with daidzin (Figure 3), indicating a recipe for the further design of inhibitors with enhanced binding for ALDH2.

### 2.6. Drug Selectivity against Human ALDH Family

ALDH enzymes have a large diversity in the amino acid sequences, whereas they tend to fold into similar structures [53]. We used the GRaphlet-based Aligner (GR-Align) [54] to align 3D structures of the 19 members in the human ALDH family (Table 5). After alignment, protein C_α_ atoms showed a RMSD of 0.1–1.0 nm between different members. For instance, the fold architecture of class 2 ALDHs (ALDH2) is close to the six members of class 1 ALDHs with a RMSD of ≤0.16 nm, and it also shows a similarity with the member A1 of classes 5–9 ALDHs (RMSD ~0.2 nm). Large RMSD values are observed for ALDH3B2, ALDH16A1, and ALDH18A1 (Table 5), indicating a discrepancy in the protein folds compared to other members.

Docking predictions showed that the chosen inhibitors of butenafine and olaparib are probably potent to other ALDHs (Table 5). Butenafine yielded an equal or stronger binding strength with ALDH1A1, ALDH1A2, ALDH3A2, ALDH7A1, and ALDH9A1 compared to that with ALDH2. A similar finding for olaparib is observed for half of the human ALDH family members. Note that the 19 ALDH members have different net charges (*q* in Table 5). Considering the issue of evaluating electrostatic interactions for the Vina docking and structural flexibility of ALDHs (mentioned above), further studies of, for instance, high accuracy calculations and in vitro experiments appeared necessary to test the selectivity of both compounds for ALDHs.

## 3. Computational Methods

### 3.1. Docking Protocol

#### 3.1.1. Ligand Preparation

Molecular structures of 1615 FDA-approved drugs were downloaded from the ZINC 15 database in the MOL2 format (https://zinc.docking.org/substances/subsets/fda (accessed on 7 December 2022)) [55]. Considering the titration states at different pH values, there were 2115 drugs in total for use as ligands in the virtual screening.

#### 3.1.2. Receptor Preparation

In order to consider the receptor flexibility, we performed molecular dynamics (MD) simulations of the ALDH2 tetramer in the apo form for 6 ns. The MD protocol was given in the following (Section 3.2). Four replicates were carried out with different initial velocities, and MD trajectories were saved every 1 ps (i.e., 6000 × 4 frames of ALDH2 monomers for each replica); one replica was extended to 30 ns for monitoring the structural stability. After discarding the first 1ns for equilibration, MD snapshots of ALDH2 monomers were extracted with an interval of 50 ps, giving rise to 1600 frames in total. Such a task was carried out by the GROMACS tool of “gmx trjconv” [56]. We concatenated these frames into a single trajectory and then clustered it with a RMSD cutoff of 0.0757 nm, using the GROMACS tools of “gmx trjcat” and “gmx cluster”, respectively [56]. Note that the cutoff threshold could be tuned by purpose and our choice resulted in 50 clusters. The middle structure for each cluster was chosen as a representative receptor configuration. Together with the crystal structure of ALDH2 (i.e., chain A of protein 2VLE), we had 51 receptors for docking calculations. Two python scripts (prepare_receptor4.py and prepare_ligand4.py) in the MGLTools (https://ccsb.scripps.edu/mgltools (accessed on 7 December 2022)) [57] were used to prepare receptors and ligands, respectively, in the PDBQT format for docking calculations.

#### 3.1.3. Docking Calculation

Virtual screening of FDA-approved drugs against ALDH2 was carried out using the Autodock Vina software (version 1.1.2) [46]. All of the 50 MD-derived receptors were aligned with the crystal structure of ALDH2 (chain A) via the least-square fitting of protein backbone atoms. This facilitated the use of a single searching space for all the receptors. Using the ALDH2/daidzin complex in the protein 2VLE as a reference, the searching space was centered roughly on the geometrical center of the inhibitor, and its size in each dimension was 3.0 nm. Other input parameters for the Vina docking were set by default. The cofactor NAD^+^ was regarded as one residue of the receptor and was involved in the binding with the inhibitors. The binding poses with the strongest binding affinity for each ligand were collected for further analysis.

### 3.2. MD Simulation of ALDH2 Tetramer

Molecular dynamics (MD) simulations of mitochondrial aldehyde dehydrogenase (ALDH2) tetramers in the apo or complex form were carried out using the GROMACS software (version 2018.4) [56]. Initial structures were taken from the Protein Data Bank (PDB ID: 2VLE; resolution: 2.4 Å), in which human ALDH2 formed a tetramer bound to the inhibitor daidzin in the absence of cofactor NAD^+^ [42]. Alignment of protein backbone atoms with the NAD^+^-bound protein 1CW3 [44] were able to produce a ALDH2 tetramer in complex with both inhibitor and cofactor. Such alignment was carried out using the GROMACS utility of “gmx confrms” [56]. The Amber 99SB-ILDN force field [58] was chosen to model ALDH2 and ions (Mg^2+^, Na^+^, and Cl^−^), and the General Amber Force Field (GAFF) [59] was used for the inhibitors. After structural optimization in gas phase at HF/6-31G* via the Gaussian 09 software [60], we calculated the restrained electrostatic potential (RESP) charges of the inhibitors using the “antechamber” tool [61]. Force field parameters of NAD^+^ were taken from previous studies [62,63] and can be downloaded freely from the group of Dr. Richard Bryce (http://research.bmh.manchester.ac.uk/bryce/amber (accessed on 7 December 2022)). The rigid TIP3P model [64] was utilized to describe water molecules.

ALDH2 tetramer was placed in the center of a cubic box with a length of 12 nm, and the distance between the protein and the box edge was roughly 1.0 nm. The box was filled with water molecules, and Na^+^ and Cl^−^ ions were then added to the simulation box via replacing water molecules randomly to neutralize the system to a physiological salt concentration of 0.15 mol/L. For the simulation of ALDH2 without cofactor and inhibitor, for instance, the system contained one ALDH2 tetramer, 198 Na^+^, 174 Cl^−^, and 54521 water molecules. After energy minimization, the systems were equilibrated by 100 ps at NVT and then by 400 ps at NPT with the position of protein backbone atoms restrained using a force constant of 1000 kJ mol^−1^ nm^−2^. Subsequently, we turned off the position restraints and performed production simulations for 30 ns at NPT (P = 1 bar and *T* = 298.15 *K*), allowing equilibration of protein structures and/or protein–ligand interactions. For more details on the simulation setup, refer to our previous work on the ALDH2 dimer [45]. All of the figures for 3D and 2D receptor–ligand interactions were generated by the Biovia Discovery studio visualizer software.

### 3.3. Toxicity Prediction

Ideal inhibitors are supposed to be potent and have little or low toxicity. The selected drugs from the virtual screening with a strong binding with ALDH2 were examined by a web platform of ProTox-II (https://tox-new.charite.de/protox_II (accessed on 7 December 2022)) [47] for toxicity evaluation. Two different levels of toxicity were tested: organ toxicity (hepatotoxicity) and toxicological endpoints (mutagenicity, carcinotoxicity, cytotoxicity and immunotoxicity).

### 3.4. MM-PBSA Analysis

After toxicity evaluation, the docked complexes of inhibitor candidates with ALDH2 (ALDH2/inhibitor/cofactor) tetramers were subjected to 30 ns MD simulations for the equilibration of receptor–ligand interactions. Only one ALDH2 monomer was adopted to implement the virtual screening to predict binding modes, and via alignment of protein backbone atoms (as stated in the Section 3.1.3), we obtained receptor–ligand complexes for the ALDH2 tetramer and used them as the initial configuration for the MD simulations.

For a production run of, for instance, the ALDH2/daidzin/NAD^+^ complex, the simulated system was composed of one ALDH2 tetramer, 4 daidzin, 4 NAD^+^, 4 Mg^2+^, and 194 Na^+^, 174 Cl^−^, and 53,584 water molecules. Water molecules and Na^+^, Cl^−^, and NAD^+^-bound Mg^2+^ ions were stripped from the production simulations, and simulation snapshots were then extracted from the last 10 ns trajectory. With an interval of 100 ps, the resulting 100 conformations of ALDH2/inhibitor/cofactor complexes were used to compute the binding energy (∆*E*_bind_) between receptor and ligand molecules from the molecular mechanics Poisson–Boltzmann surface area (MM-PBSA) analysis (Equation (2)).

(2) $$∆E_{\mathrm{bind}}=∆E_{\mathrm{MM}}+∆G_{\mathrm{polar}}+∆G_{\mathrm{nonpolar}}$$

where $∆E_{\mathrm{MM}}$ is the molecular mechanics (MM) part including the contributions from van der Waals (∆*E*_vdW_) and electrostatic (∆*E*_elec_) interactions. ∆*G*_polar_ and ∆*G*_nonpolar_ are the polar and nonpolar solvation contributions. Such a task was accomplished by the “g_mmpbsa” toolkit [50]. Note that the results from eq 2 are binding energies and adding an entropy part (−*T*∆S) would give rise to binding free energies. A module for the entropy calculation was not yet incorporated into the “g_mmpbsa” toolkit. ∆*G*_polar_ was computed by the built-in APBS software [65], and a solvent accessible surface area (SASA) model [50] was utilized to calculate ∆*G*_nonpolar_. The scripts (MmPbSaStat.py and MmPbSaDecomp.py) downloaded from http://rashmikumari.github.io/g_mmpbsa/Usage.html (accessed on 7 December 2022) were used for the energy statistics and decompositions in the MM-PBSA analysis.

### 3.5. Inhibition against Different ALDHs

The 19 NAD(P)^+^-dependent members in the human ALDH superfamily were chosen to evaluate the selectivity of inhibitor candidates via docking predictions. Receptor structures were extracted for the Protein Data Bank (PDB) or AlphaFold Protein Structure Database (https://alphafold.ebi.ac.uk/ (accessed on 7 December 2022)) [66]; the former included the predicted structures in the latter as well. The members in the ALDH family show a similar architecture with three domains for substrate binding, cofactor binding, and oligomerization (Figure 1) [53]. Alignment with the ALDH2/daidzin/NAD^+^ complex therefore allows a determination of the substrate/cofactor binding regions of ALDHs, and one can define the search space in the docking then. The alignment of protein 3D structures was carried out by the GR-Align software (version 1.5) with the graphlet degree similarity [54]. Similar search space and docking protocol were used for the docking calculations, as mentioned above.

## 4. Conclusions

Through virtual screening of FDA-approved drugs, toxicity evaluation, molecular dynamics (MD) simulation of ALDH2–inhibitor complexes, and MM-PBSA analysis on the binding energies, we showed that butenafine (net charge *q* = +1 *e*) and olaparib (*q* = 0) can be potent inhibitors against ALDH2. Binding energies (Δ*E*_bind_) for these two compounds were −313.4 and −127.6 kJ/mol, respectively; the binding strengths were comparable to or stronger than the previously reported inhibitors of daidzin (Δ*E*_bind_ = −110.1 kJ/mol) and CVT-10216 (Δ*E*_bind_ = −132.3 kJ/mol) [15,26,31,45]. The strong binding of butenafine was mainly ascribed to its positive charge offering electrostatic attraction with the negative receptor (*q* = −6 *e* for ALDH2 plus NAD^+^). Several negatively charged compounds were also hit from the screening with the Autodock Vina software. However, the MM-PBSA analysis demonstrated that owing to electrostatic repulsion, these compounds were unfavorable in the thermodynamics of binding with ALDH2. This pointed out an issue of the Vina scoring in the evaluation of strong charge–charge interactions. Because the Vina scoring, as designed, did not use atomic charges to deal with electrostatic interactions, the scoring results should be used with care, in particular for highly charged molecules. Further verification of the selected compounds from high-accuracy predictions and experimental measurements is necessary to test the enzyme inhibition activity and selectivity. The identified key residues responsible for ALDH2–inhibitor associations indicated that there is still room for the design of ALDH2 inhibitors with enhanced binding via, for instance, a rational use of the interacting residues at both ends of the substrate-binding hydrophobic tunnel of the receptor ALDH2.

## Figures and Tables

**Table 1:** Top 10 compounds from virtual screening of FDA-approved drugs via Autodock Vina using the crystal structure of ALDH2 as receptor and the toxicity prediction via ProTox-II.

ZINC ID	Name	Molecular Structure	*q*	∆*E*_dock_	Toxicity				
					Dili	Carcino	Immuno	Mutagen	Cyto
ZINC003784182	differin	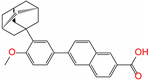	−1	−48.5	N(83)	Y(61)	N(85)	N(73)	N(76)
ZINC011679756	eltrombopag	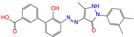	−3	−46.9	Y(67)	N(57)	N(72)	N(56)	N(84)
ZINC003824921	fexofenadine	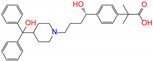	0	−45.6	N(99)	Y(50)	N(86)	N(85)	N(81)
ZINC035801098	indacaterol	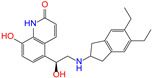	0	−45.6	N(74)	N(60)	Y(80)	N(61)	N(66)
ZINC003831151	montelukast	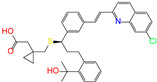	−1	−45.6	N(56)	N(64)	N(67)	N(72)	N(67)
ZINC019632618	imatinib	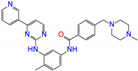	1	−45.2	Y(71)	N(67)	Y(66)	N(73)	N(52)
ZINC004097343	itc	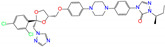	0	−45.2	Y(88)	N(62)	Y(75)	N(53)	N(90)
ZINC006745272	stivarga	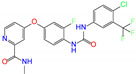	0	−45.2	Y(82)	N(50)	Y(99)	N(79)	Y(77)
ZINC000643138	nizoral	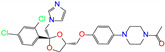	1	−43.9	Y(76)	N(51)	Y(98)	N(69)	N(63)
ZINC000537791	amaryl	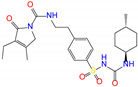	−1	−43.5	N(74)	N(61)	N(98)	N(75)	N(68)
	CVT-10216	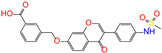	0	−43.5	Y(56)	N(60)	N(95)	Y(54)	N(66)
	daidzin	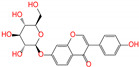	0	−38.1	N(82)	N(85)	N(59)	N(76)	N(69)

ZINC ID and name are the compound ID and name in the ZINC database, respectively. Two isoflavone analogues of CVT-10216 (IC_50_ = 0.029 μM) and daidzin (IC_50_ = 0.08 μM) with potent inhibition against ALDH2 are also listed for comparison. *q* is the net charge of compounds. ∆*E*_dock_ is the binding affinity from docking calculations in units of kJ/mol. Toxicity predictions include hepatotoxicity (dili for short), carcinogenicity (carcino), immunotoxicity (immuno), mutagenicity (mutagen), and cytotoxicity (cyto). N means inactive, and Y is active. The confidence (%) for the toxicity prediction is given in parenthesis. Differin, fexofenadine, and daidzin are selected for the subsequent MD simulation and MM-PBSA analysis.

**Table 2:** Top 10 compounds from virtual screening of FDA-approved drugs via Autodock Vina using MD-derived structures of ALDH2 as receptor and the toxicity prediction via ProTox-II.

ZINC ID	Name	Molecular Structure	*q*	∆*E*_dock_	Toxicity				
					Dili	Carcino	Immuno	Mutagen	Cyto
ZINC100017856	mepron	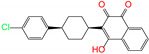	−1	−51.1	N(64)	N(53)	N(86)	N(53)	N(87)
ZINC003784182	differin	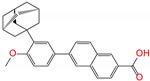	−1	−48.5	N(83)	Y(61)	N(85)	N(73)	N(76)
ZINC028639340	noxafil	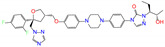	0	−47.7	Y(86)	N(62)	Y(99)	N(56)	N(75)
ZINC000896717	accolate	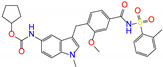	−1	−47.3	Y(76)	N(57)	N(65)	N(67)	N(56)
ZINC003938482	noxafil	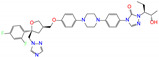	0	−47.3	Y(86)	N(62)	Y(99)	N(56)	N(75)
ZINC004175630	orap	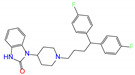	1	−47.3	N(78)	N(69)	Y(89)	N(86)	N(65)
ZINC040430143	olaparib	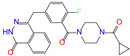	0	−46.9	N(62)	N(57)	N(95)	N(54)	N(65)
ZINC011679756	eltrombopag	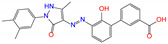	−2	−46.9	Y(67)	N(57)	N(72)	N(56)	N(84)
ZINC001530975	butenafine	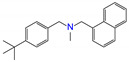	1	−46.9	N(85)	N(57)	N(92)	N(71)	N(75)
ZINC000057255	thalitone	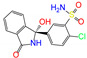	0	−46.4	N(79)	N(71)	Y(80)	N(77)	N(67)

Refer to the footnotes of Table 1 for the details of compounds and toxicity predictions. Mepron, differin, olaparib, and butenafine are selected for the subsequent MD simulation and MM-PBSA analysis.

**Table 3:** Time-averaged root-mean-square deviation (RMSD) of protein backbone atoms and receptor-ligand binding energies (Δ*E*_bind_) for the ALDH2 tetramer and monomers.

Compound	Chain A	Chain B	Chain C	Chain D	Tetramer
	RMSD (nm)				
butenafine	0.13	0.10	0.09	0.10	0.15
olaparib	0.10	0.10	0.10	0.09	0.13
fexofenadine	0.12	0.10	0.11	0.11	0.14
mepron	0.12	0.11	0.10	0.10	0.13
differin	0.10	0.10	0.09	0.10	0.11
daidzin	0.09	0.11	0.10	0.09	0.12
free	0.10	0.10	0.12	0.11	0.13
**Compound**	**Chain A**	**Chain B**	**Chain C**	**Chain D**	**<Δ*E*_bind_>**
	Δ*E*_bind_ (kJ/mol)				
butenafine	−290.0 ± 3.9	−306.9 ± 3.2	−313.4 ± 3.1	−270.2 ± 6.0	−313.0 ± 4.2
olaparib	−119.2 ± 2.4	−115.1 ± 3.3	−127.6 ± 8.7	−121.9 ± 5.1	−126.8 ± 5.4
fexofenadine	−35.2 ± 10.4	−94.4 ± 5.2	−34.2 ± 13.9	−90.1 ± 5.2	−93.8 ± 9.4
mepron	35.0 ± 3.5	8.3 ± 1.1	47.5 ± 5.5	44.4 ± 4.0	8.3 ± 3.9
differin	64.2 ± 6.3	58.9 ± 5.3	92.7 ± 7.8	70.9 ± 6.9	59.5 ± 6.6
daidzin	−79.5 ± 9.9	−91.9 ± 4.5	−110.1 ± 4.6	−86.9 ± 4.9	−110.1 ± 6.4

The last 10 ns trajectories were used to calculate RMSD and Δ*E*_bind_. Standard deviations of the RMSDs are smaller than 0.01 nm. The RMSD of ALDH2 in the ligand-free (apo) form is given as well for comparison. The trajectories were divided into five blocks for improved statistics on the energies via block averaging. <Δ*E*_bind_> is the averaged binding energy weighted by their Boltzmann factors (Equation (1)).

**Table 4:** Decomposition of binding energies (kJ/mol) of the selected inhibitors with ALDH2 via MM-PBSA using simulation trajectories.

Compound	*q*	Δ*E*_vdW_	Δ*E*_elec_	Δ*E*_MM_	Δ*G*_polar_	Δ*G*_nonpolar_	Δ*G*_sol_	Δ*E*_bind_
butenafine	+1	−220.4 ± 2.2	−280.6 ± 1.3	−501.0 ± 1.9	207.6 ± 2.3	−20.0 ± 0.1	187.6 ± 2.4	−313.4 ± 3.1
olaparib	0	−232.2 ± 4.9	−8.6 ± 2.6	−240.8 ± 7.4	134.9 ± 3.1	−21.7 ± 0.4	113.2 ± 3.2	−127.6 ± 8.7
fexofenadine	0	−216.9 ± 2.7	−183.6 ± 5.4	−400.4 ± 7.0	330.7 ± 8.6	−24.7 ± 0.4	306.0 ± 8.5	−94.4 ± 5.2
mepron	−1	−227.5 ± 1.1	101.9 ± 5.0	−125.5 ± 4.0	152.9 ± 4.0	−19.1 ± 0.1	133.8 ± 3.9	8.3 ± 1.1
differin	−1	−199.5 ± 3.6	74.8 ± 4.1	−124.7 ± 1.5	203.4 ± 5.0	−19.8 ± 0.2	183.6 ± 5.2	58.9 ± 5.3
daidzin	0	−222.9 ± 3.6	−65.1 ± 2.5	−288.1 ± 3.5	197.7 ± 3.9	−19.8 ± 0.2	177.9 ± 3.8	−110.1 ± 4.6

The energies correspond to the monomer with the lowest binding affinity (see Table 3). *q* is the formal charge (*e*) of the compounds, and the net charge of ALDH2 monomer is −6 *e*. Δ*E*_MM_ is the MM part and amounts to Δ*E*_vdW_ + Δ*E*_elec_. Δ*G*_sol_ is the solvation energies (Δ*G*_polar_ + Δ*G*_nonpolar_). For each compound, 100 frames from the last 10 ns trajectories were used for the MM-PBSA analysis. Block averaging were used for the energy calculations for improved statistics.

**Table 5:** Structural comparison of 19 NAD(P)^+^-dependent members in the hum an ALDH family and their binding affinities with butenafine and olaparib from docking predictions.

Name	Entry	Identifier	Residues	*q*	RMSD (nm)		∆*E*_dock_ (kJ/mol)	
					Others	ALDH2	Butenafine	Olaparib
ALDH1A1	P00352	4WB9	21–501	−2	0.26 ± 0.18	0.13	**−40.3 ± 0.2**	**−42.7 ± 0.2**
ALDH1A2	O94788	6B5G	38–518	−4	0.27 ± 0.19	0.12	**−39.3 ± 0.1**	**−45.3 ± 1.7**
ALDH1A3	P47895	5FHZ	32–507	−2	0.26 ± 0.18	0.15	−36.6 ± 0.2	**−39.7 ± 0.1**
ALDH1B1	P30837	7RAD	37–500	−5	0.29 ± 0.18	0.04	−37.6 ± 0.2	−36.8 ± 0.9
ALDH1L1	O75891	AF-O75891-F1	422–902	−2	0.26 ± 0.17	0.16	−28.5 ± 0.1	−36.4 ± 0.1
ALDH1L2	Q3SY69	AF-Q3SY69-F1	443–923	−4	0.26 ± 0.17	0.16	−27.5 ± 0.2	−34.3 ± 0.1
ALDH2	P05091	2VLE	37–500	−5	0.30 ± 0.23	0.00	−39.0 ± 3.9	−39.2 ± 2.6
ALDH3A1	P30838	4L2O	1–432	−6	0.37 ± 0.22	0.35	−35.3 ± 0.3	−35.5 ± 0.3
ALDH3A2	P51648	4QGK	1–432	−3	0.37 ± 0.21	0.35	**−40.5 ± 1.7**	**−41.4 ± 1.2**
ALDH3B1	P43353	AF-P43353-F1	1–432	−2	0.38 ± 0.22	0.33	−34.6 ± 0.2	−34.2 ± 1.4
ALDH3B2	P48448	AF-P48448-F1	1–353	−7	0.68 ± 0.30	0.85	−35.6 ± 0.4	−37.9 ± 0.2
ALDH4A1	P30038	4OE5	64–551	−4	0.49 ± 0.18	0.48	−34.6 ± 0.4	**−41.8 ± 0.1**
ALDH5A1	P51649	2W8R	61–535	−2	0.27 ± 0.15	0.19	−33.5 ± 0.1	**−40.6 ± 0.1**
ALDH6A1	Q02252	AF-Q02252-F1	39–517	4	0.32 ± 0.21	0.20	−28.4 ± 0.2	−33.5 ± 0.6
ALDH7A1	P49419	4ZUK	51–500	2	0.31 ± 0.14	0.18	**−41.2 ± 1.6**	**−46.4 ± 0.1**
ALDH8A1	Q9H2A2	AF-Q9H2A2-F1	10–487	−1	0.31 ± 0.20	0.21	−25.8 ± 0.4	−31.4 ± 0.1
ALDH9A1	P49189	6VR6	12–488	−3	0.30 ± 0.21	0.16	**−41.2 ± 0.2**	**−43.7 ± 0.9**
ALDH16A1	Q8IZ83	AF-Q8IZ83-F1	26–488	−9	0.61 ± 0.33	0.56	−30.5 ± 0.1	−37.2 ± 0.1
ALDH18A1	P54886	2H5G	361–770	−7	0.81 ± 0.19	0.80	−29.5 ± 0.6	**−40.1 ± 0.4**

Sequence entries in the UniProt database and structure identifies in the PDB (four-letter codes) or AlphaFold protein structure database (with a prefix of AF) were given for the 19 ALDHs. *q* is the net charge of the proteins in the absence of cofactor. RMSD is the root-mean-square deviation of protein C_α_ atoms of ALDH relative to other members (averaged over 18 pairs) or to ALDH2 after structural alignment via the GR-Align software. Binding energies (∆*E*_dock_) are averaged over 100 replicates of docking calculations with random seeds. The energies that are more negative (i.e., a stronger binding) than that of ALDHs with butenafine or olaparib are marked in bold.

## Data Availability

Data is contained within the article.

## Supplementary Materials

The following supporting information can be downloaded at: https://www.mdpi.com/article/10.3390/molecules27248773/s1, Energy decomposition for identified key residues with a large contribution to the ALDH2 binding with butenafine (Table S1), olaparib (Table S2), fexofenadine (Table S3), and daidzin (Table S4).
