# Supplementary material for: Virtual Screening of FDA-Approved Drugs for Enhanced Binding with Mitochondrial Aldehyde Dehydrogenase
Source: Molecules. 2022 Dec 10;27(24):8773. doi: 10.3390/molecules27248773 (PMC9781114; doi:10.3390/molecules27248773)
Supplement: Supplementary file 1 [file molecules-27-08773-s001.zip › molecules-2067750-supplementary.pdf]

Supplementary Materials for

Virtual Screening of FDA-Approved Drugs for Enhanced Binding with Mitochondrial Aldehyde Dehydrogenase

Boqian Zhou [1,2], Yongguang Zhang [1], Wanyun Jiang [1] and HaiyangZhang [1,*]

[1] Department of Biological Science and Engineering, School of Chemistry and Biological Engineering, University of Science and Technology Beijing, Beijing 100083, China; 3120221410@bit.edu.cn (B.Z.); zhangyg0507@sina.com (Y.Z.); m202110881@xs.ustb.edu.cn (W.J.)

[2] School of Life Sciences, Beijing Institute of Technology, Beijing 100081, China

* Correspondence: zhanghy@ustb.edu.cn

Table S1. Energy decomposition (kJ/mol) for identified key residues with a large contribution to the ALDH2 binding with butenafine

Residue	ΔE_{MM}	$\Delta E_{\mathrm{polar}}$	$\Delta E_{\mathrm{nonpolar}}$	ΔE_{bind}
GLU-96	-15.4 ± 0.0	1.9 ± 0.0	0.0 ± 0.0	-13.6 ± 0.0
ARG-99	18.2 ± 0.0	-3.2 ± 0.0	0.0 ± 0.0	15.0 ± 0.0
GLU-106	-26.8 ± 0.1	8.7 ± 0.1	0.0 ± 0.0	-18.1 ± 0.1
ASP-109	-19.1 ± 0.0	4.7 ± 0.0	0.0 ± 0.0	-14.4 ± 0.0
LYS-112	32.3 ± 0.1	-11.9 ± 0.2	0.0 ± 0.0	20.4 ± 0.2
ASP-121	-35.8 ± 0.1	16.1 ± 0.5	-0.0 ± 0.0	-19.8 ± 0.4
ASP-123	-23.6 ± 0.1	4.1 ± 0.1	0.0 ± 0.0	-19.5 ± 0.1
LYS-127	25.2 ± 0.2	-4.1 ± 0.1	-0.0 ± 0.0	21.1 ± 0.1
ARG-130	17.3 ± 0.0	-2.9 ± 0.0	0.0 ± 0.0	14.4 ± 0.0
LYS-138	13.9 ± 0.0	-1.2 ± 0.0	0.0 ± 0.0	12.6 ± 0.0
LYS-178	40.3 ± 0.1	-19.2 ± 0.2	0.0 ± 0.0	21.1 ± 0.2
LYS-192	18.6 ± 0.0	-4.4 ± 0.0	0.0 ± 0.0	14.2 ± 0.0
GLU-195	-18.5 ± 0.0	4.4 ± 0.0	0.0 ± 0.0	-14.1 ± 0.0
LYS-240	22.9 ± 0.0	-5.6 ± 0.1	0.0 ± 0.0	17.3 ± 0.1
GLU-248	-17.3 ± 0.1	2.2 ± 0.0	0.0 ± 0.0	-15.1 ± 0.0
ARG-251	16.6 ± 0.0	-1.8 ± 0.0	0.0 ± 0.0	14.7 ± 0.0
ARG-264	14.3 ± 0.0	-1.4 ± 0.0	0.0 ± 0.0	12.9 ± 0.0
GLU-268	-51.4 ± 0.2	30.8 ± 0.5	-0.6 ± 0.0	-21.2 ± 0.4
LYS-272	20.6 ± 0.0	-3.4 ± 0.1	0.0 ± 0.0	17.2 ± 0.0
GLU-288	-16.9 ± 0.0	1.8 ± 0.0	0.0 ± 0.0	-15.1 ± 0.0
ARG-307	17.9 ± 0.1	-3.4 ± 0.0	0.0 ± 0.0	14.6 ± 0.0
ARG-325	15.4 ± 0.0	-1.5 ± 0.0	0.0 ± 0.0	14.0 ± 0.0
ARG-329	20.0 ± 0.0	-3.7 ± 0.0	0.0 ± 0.0	16.3 ± 0.0
GLU-340	-17.0 ± 0.0	1.9 ± 0.0	0.0 ± 0.0	-15.0 ± 0.0
ASP-346	-17.3 ± 0.1	3.2 ± 0.0	0.0 ± 0.0	-14.0 ± 0.0
LYS-352	18.3 ± 0.1	-3.1 ± 0.1	0.0 ± 0.0	15.3 ± 0.0
LYS-397	13.6 ± 0.1	-0.8 ± 0.0	0.0 ± 0.0	12.8 ± 0.0
GLU-398	-17.0 ± 0.1	1.6 ± 0.0	0.0 ± 0.0	-15.4 ± 0.1
GLU-399	-31.4 ± 0.1	11.1 ± 0.1	0.0 ± 0.0	-20.3 ± 0.1
ASP-457	-42.4 ± 0.2	17.4 ± 0.6	-0.4 ± 0.0	-25.4 ± 0.5
LYS-469	16.6 ± 0.1	-1.9 ± 0.1	0.0 ± 0.0	14.7 ± 0.0
ARG-475	30.7 ± 0.1	-10.2 ± 0.1	-0.0 ± 0.0	20.5 ± 0.1
GLU-476	-42.2 ± 0.2	17.4 ± 0.2	-0.1 ± 0.0	-24.9 ± 0.1
GLU-479	-17.0 ± 0.0	2.2 ± 0.0	0.0 ± 0.0	-14.8 ± 0.0
GLU-487	-14.1 ± 0.0	1.4 ± 0.0	0.0 ± 0.0	-12.7 ± 0.0
NDP-501	-18.7 ± 0.1	7.5 ± 0.1	-0.2 ± 0.0	-11.4 ± 0.2

Table S2. Energy decomposition (kJ/mol) for identified key residues with a large contribution to the ALDH2 binding with olaparib

Residue	ΔE_{MM}	ΔE_{polar}	$\Delta E_{\text{nonpolar}}$	ΔE_{bind}
ALA-7	-0.0 ± 0.0	0.3 ± 0.0	0.0 ± 0.0	0.3 ± 0.0
ARG-99	-0.0 ± 0.0	1.8 ± 0.1	0.0 ± 0.0	1.8 ± 0.0
LYS-112	-0.6 ± 0.1	1.2 ± 0.2	0.0 ± 0.0	0.6 ± 0.2
VAL-120	-7.3 ± 0.2	0.9 ± 0.1	-1.0 ± 0.0	-7.4 ± 0.2
ASP-121	0.0 ± 0.1	3.1 ± 0.6	-0.0 ± 0.0	3.1 ± 0.5
ASP-123	-2.1 ± 0.1	-2.0 ± 0.3	-0.0 ± 0.0	-4.1 ± 0.2
MET-124	-6.0 ± 0.2	1.1 ± 0.1	-0.8 ± 0.0	-5.7 ± 0.1
LYS-127	1.1 ± 0.1	4.0 ± 0.2	-0.0 ± 0.0	5.1 ± 0.2
PHE-170	-5.7 ± 0.1	1.5 ± 0.0	-0.4 ± 0.0	-4.6 ± 0.1
TRP-177	-17.2 ± 0.2	8.4 ± 0.1	-1.4 ± 0.0	-10.2 ± 0.2
LYS-178	-7.7 ± 0.1	8.0 ± 0.4	-0.1 ± 0.0	0.1 ± 0.4
GLU-268	4.1 ± 0.1	1.7 ± 0.4	-0.1 ± 0.0	5.6 ± 0.4
ASP-282	-0.3 ± 0.0	-0.2 ± 0.0	0.0 ± 0.0	-0.6 ± 0.0
ASP-284	-0.1 ± 0.0	-0.3 ± 0.0	0.0 ± 0.0	-0.4 ± 0.0
GLU-288	0.2 ± 0.0	-1.1 ± 0.0	0.0 ± 0.0	-0.8 ± 0.1
PHE-292	-0.3 ± 0.0	-0.4 ± 0.0	-0.0 ± 0.0	-0.7 ± 0.0
PHE-296	-5.1 ± 0.2	1.9 ± 0.1	-0.5 ± 0.0	-3.7 ± 0.2
CYS-301	-0.2 ± 0.0	1.7 ± 0.1	-0.0 ± 0.0	1.5 ± 0.1
CYS-302	-4.4 ± 0.1	4.4 ± 0.1	-0.4 ± 0.0	-0.4 ± 0.1
CYS-303	-2.4 ± 0.1	1.2 ± 0.1	-0.1 ± 0.0	-1.3 ± 0.1
ARG-325	-0.1 ± 0.0	0.7 ± 0.0	0.0 ± 0.0	0.6 ± 0.0
ARG-329	-1.2 ± 0.0	2.4 ± 0.1	0.0 ± 0.0	1.3 ± 0.1
GLU-340	0.8 ± 0.0	-1.3 ± 0.0	0.0 ± 0.0	-0.5 ± 0.0
ASP-457	-9.8 ± 0.3	6.9 ± 0.6	-0.7 ± 0.0	-3.6 ± 0.5
PHE-459	-15.2 ± 0.2	4.2 ± 0.1	-1.3 ± 0.0	-12.3 ± 0.2
PHE-465	-13.3 ± 0.2	3.2 ± 0.0	-0.7 ± 0.0	-10.9 ± 0.2
NDP-501	0.0 ± 0.0	0.6 ± 0.1	-0.0 ± 0.0	0.5 ± 0.1

Table S3. Energy decomposition (kJ/mol) for identified key residues with a large contribution to the ALDH2 binding with fexofenadine

Residue	ΔE_{MM}	$\Delta E_{\mathrm{polar}}$	$\Delta E_{\mathrm{nonpolar}}$	ΔE_{bind}
ALA-7	4.8 ± 0.1	0.1 ± 0.0	0.0 ± 0.0	4.9 ± 0.1
ARG-99	5.2 ± 0.1	0.4 ± 0.0	0.0 ± 0.0	5.5 ± 0.0
LYS-112	-3.3 ± 0.2	5.0 ± 0.2	0.0 ± 0.0	1.7 ± 0.2
VAL-120	-5.1 ± 0.1	-0.5 ± 0.1	-1.0 ± 0.0	-6.6 ± 0.1
ASP-121	-5.4 ± 0.3	2.3 ± 0.6	-0.0 ± 0.0	-3.1 ± 0.5
ASP-123	-9.1 ± 0.1	-0.8 ± 0.1	0.0 ± 0.0	-9.8 ± 0.1
MET-124	-3.0 ± 0.2	0.8 ± 0.1	-0.5 ± 0.0	-2.7 ± 0.1
LYS-127	10.1 ± 0.2	0.8 ± 0.1	0.0 ± 0.0	10.9 ± 0.1
PHE-170	-9.5 ± 0.1	2.8 ± 0.1	-0.6 ± 0.0	-7.2 ± 0.1
TRP-177	-2.9 ± 0.1	1.1 ± 0.0	-0.2 ± 0.0	-2.0 ± 0.1
LYS-178	-10.6 ± 0.1	2.9 ± 0.1	0.0 ± 0.0	-7.7 ± 0.1
GLU-268	12.4 ± 0.1	-2.3 ± 0.2	-0.1 ± 0.0	10.0 ± 0.2
ASP-282	-5.0 ± 0.0	0.4 ± 0.0	0.0 ± 0.0	-4.6 ± 0.0
ASP-284	-5.5 ± 0.1	0.7 ± 0.0	0.0 ± 0.0	-4.9 ± 0.1
GLU-288	-15.0 ± 0.2	8.4 ± 0.3	-0.2 ± 0.0	-6.7 ± 0.2
PHE-292	-7.3 ± 0.2	2.7 ± 0.1	-1.1 ± 0.0	-5.6 ± 0.2
PHE-296	-10.8 ± 0.2	3.8 ± 0.1	-1.1 ± 0.0	-8.1 ± 0.2
CYS-301	-20.9 ± 0.2	11.7 ± 0.1	-0.5 ± 0.0	-9.7 ± 0.1
CYS-302	-19.0 ± 0.2	13.9 ± 0.1	-0.4 ± 0.0	-5.5 ± 0.2
CYS-303	-13.0 ± 0.4	7.9 ± 0.2	-0.2 ± 0.0	-5.3 ± 0.3
ARG-325	7.0 ± 0.1	-1.3 ± 0.0	0.0 ± 0.0	5.7 ± 0.0
ARG-329	8.0 ± 0.1	2.2 ± 0.3	-0.0 ± 0.0	10.2 ± 0.3
GLU-340	-8.8 ± 0.1	0.4 ± 0.1	-0.0 ± 0.0	-8.4 ± 0.1
ASP-457	-31.4 ± 0.3	35.0 ± 0.6	-0.9 ± 0.0	2.8 ± 0.5
PHE-459	-9.2 ± 0.2	2.6 ± 0.1	-0.7 ± 0.0	-7.3 ± 0.2
PHE-465	-3.9 ± 0.1	2.5 ± 0.0	-0.3 ± 0.0	-1.7 ± 0.1
NDP-501	1.3 ± 0.2	0.8 ± 0.2	-0.0 ± 0.0	2.1 ± 0.1

Table S4. Energy decomposition (kJ/mol) for identified key residues with a large contribution to the ALDH2 binding with daidzin

Residue	ΔE_{MM}	ΔE_{polar}	$\Delta E_{nonpolar}$	ΔE_{bind}
ALA-7	-0.5 ± 0.0	0.2 ± 0.0	0.0 ± 0.0	-0.3 ± 0.0
ARG-99	-1.5 ± 0.1	2.5 ± 0.1	0.0 ± 0.0	0.9 ± 0.1
LYS-112	-1.0 ± 0.1	5.1 ± 0.1	0.0 ± 0.0	4.1 ± 0.1
VAL-120	-5.5 ± 0.2	0.7 ± 0.0	-0.6 ± 0.0	-5.4 ± 0.1
ASP-121	0.6 ± 0.1	-5.6 ± 0.2	0.0 ± 0.0	-5.0 ± 0.1
ASP-123	3.8 ± 0.3	-4.9 ± 0.3	-0.1 ± 0.0	-1.1 ± 0.2
MET-124	-6.5 ± 0.1	2.5 ± 0.0	-0.5 ± 0.0	-4.5 ± 0.1
LYS-127	-4.3 ± 0.3	7.0 ± 0.4	-0.0 ± 0.0	2.6 ± 0.2
PHE-170	-7.0 ± 0.1	3.1 ± 0.1	-0.5 ± 0.0	-4.5 ± 0.1
TRP-177	-4.6 ± 0.1	2.0 ± 0.1	-0.2 ± 0.0	-2.7 ± 0.1
LYS-178	-0.3 ± 0.1	-0.7 ± 0.1	0.0 ± 0.0	-1.0 ± 0.1
GLU-268	-1.6 ± 0.1	0.7 ± 0.2	-0.1 ± 0.0	-1.0 ± 0.2
ASP-282	-0.6 ± 0.0	0.1 ± 0.0	0.0 ± 0.0	-0.5 ± 0.0
ASP-284	-0.6 ± 0.0	0.1 ± 0.0	0.0 ± 0.0	-0.5 ± 0.0
GLU-288	-1.1 ± 0.1	0.2 ± 0.1	0.0 ± 0.0	-0.9 ± 0.1
PHE-292	-2.9 ± 0.1	3.9 ± 0.2	-0.4 ± 0.0	0.6 ± 0.1
PHE-296	-9.8 ± 0.1	3.2 ± 0.1	-0.8 ± 0.0	-7.4 ± 0.1
CYS-301	-7.2 ± 0.1	7.0 ± 0.1	-0.2 ± 0.0	-0.4 ± 0.1
CYS-302	-6.5 ± 0.2	7.5 ± 0.1	-0.2 ± 0.0	0.7 ± 0.1
CYS-303	-8.3 ± 0.2	4.1 ± 0.1	-0.4 ± 0.0	-4.6 ± 0.2
ARG-325	0.7 ± 0.1	-0.1 ± 0.0	0.0 ± 0.0	0.6 ± 0.0
ARG-329	0.8 ± 0.2	1.3 ± 0.1	0.0 ± 0.0	2.1 ± 0.1
GLU-340	-0.3 ± 0.1	-1.0 ± 0.1	0.0 ± 0.0	-1.3 ± 0.1
ASP-457	-23.8 ± 0.3	37.1 ± 0.5	-1.1 ± 0.0	12.2 ± 0.4
PHE-459	-12.0 ± 0.2	3.4 ± 0.1	-0.8 ± 0.0	-9.3 ± 0.2
PHE-465	-5.9 ± 0.1	2.1 ± 0.0	-0.3 ± 0.0	-4.1 ± 0.1
NDP-501	-3.5 ± 0.1	4.4 ± 0.1	-0.3 ± 0.0	0.7 ± 0.2
